# On the road to sustainability – application of metallic nanoparticles obtained by green synthesis in dentistry: a scoping review

**DOI:** 10.3762/bjnano.16.128

**Published:** 2025-10-22

**Authors:** Lorena Pinheiro Vasconcelos Silva, Joice Catiane Soares Martins, Israel Luís Carvalho Diniz, Júlio Abreu Miranda, Danilo Rodrigues de Souza, Éverton do Nascimento Alencar, Moan Jéfter Fernandes Costa, Pedro Henrique Sette-de-Souza

**Affiliations:** 1 Programa de Pós-Graduação em Odontologia, Universidade de Pernambuco, Av. Norte Miguel Arraes de Alencar, 80 – Zip Code: 52071-035 - Santo Amaro, Recife/PE, Brazil; 2 Programa de Pós-Graduação em Saúde e Desenvolvimento Socioambiental, Universidade de Pernambuco, R. Cap. Pedro Rodrigues, 105 – Zip Code: 55294-902 - São José, Garanhuns/PE, Brazil; 3 Graduate Program in Health Sciences, Federal University of Rio Grande do Norte, R. Gen. Gustavo Cordeiro de Farias, s/n – Zip Code: 59078-970 - Natal/RN, Brazil; 4 Programa de Pós-Graduação em Ciências dos Materiais, Universidade Federal do Oeste da Bahia, Rua da Prainha, 1326 – Zip Code: 47805-100 - Morada Nobre, Barreiras/BA, Brazil; 5 Programa de Pós-Graduação em Química Pura e Aplicada, Universidade Federal do Oeste da Bahia, Rua da Prainha, 1326 – Zip Code: 47805-100 - Morada Nobre, Barreiras/BA, Brazil; 6 Laboratory of Micro and Nanostructured Systems, College of Pharmaceutical Sciences, Food and Nutrition, Federal University of Mato Grosso do Sul, Avenida Costa e Silva, s/n – Zip Code: 79070-900 – Bairro Universitário, Campo Grande/MS, Brazil

**Keywords:** dentistry, green chemistry technology, metal nanoparticles, nanotechnology, sustainable development

## Abstract

The growing interest in green-synthesized metallic nanoparticles reflects a global shift toward sustainable, eco-friendly technologies in biomedical innovation, particularly in dentistry. This scoping review examines the rising focus on these nanoparticles regarding their antimicrobial, regenerative, and therapeutic potential in dental applications. Among the metals studied, silver and zinc oxide nanoparticles dominate because of their broad-spectrum antimicrobial activity and enhanced biocompatibility, achieved through phytochemically mediated synthesis. Conventional nanoparticle production often relies on toxic reagents and energy-intensive processes, posing environmental and clinical challenges. In contrast, green synthesis, using plant extracts, fungi, or bacteria, offers a sustainable alternative by leveraging natural reducing agents like polyphenols and flavonoids. These bioactive compounds not only facilitate nanoparticle formation but also improve stability and biological efficacy, making them ideal for dental applications such as caries prevention, endodontic disinfection, and periodontal regeneration. Our analysis of 98 studies reveals India as the leading contributor (78.6%), driven by its rich biodiversity and strong research infrastructure. Key plant families including Lamiaceae and Fabaceae were frequently employed due to their high phenolic content. Despite promising results, gaps remain, such as the predominance of in vitro studies (68.7%) and insufficient cytotoxicity assessments (47.8%), underscoring the need for translational research. This review highlights the transformative potential of green-synthesized nanoparticles in dentistry, merging technological advancement with ecological responsibility. Future work should prioritize clinical trials, long-term safety evaluations, and standardized protocols to fully realize their therapeutic benefits.

## Introduction

Nanotechnology is an interdisciplinary field of science that involves the manipulation of materials at the nanoscale, typically ranging from 1 to 100 nm in inorganic nanomaterials, to generate structures with unique physicochemical properties [1–3]. Among the most widely studied nanomaterials are metallic nanoparticles, particularly silver (AgNPs), gold (AuNPs), and copper (CuNPs), and various metal oxide nanoparticles such as zinc oxide (ZnO-NPs), due to their high surface-to-volume ratio, chemical stability, and distinctive optical and antimicrobial properties [4–6]. These nanomaterials have been successfully applied in diverse fields, such as medicine, agriculture, cosmetics, electronics, the food industry, and, more recently, dentistry [5–6]. However, conventional chemical and physical synthesis routes often involve toxic organic solvents, high energy consumption, and hazardous reducing agents; also, they result in environmental waste, in addition to producing nanoparticles that may be toxic and poorly biocompatible [3,7].

Given the limitations of conventional synthesis methods, the green synthesis of nanoparticles has emerged as a sustainable, safe, and economically viable alternative [8–9]. This approach employs biological agents, such as plant extracts, fungi, bacteria, and algae, which contain bioactive compounds capable of acting as reducing and stabilizing agents in the formation of metallic nanoparticles [10–11]. In the case of plant extracts, several compounds, such as phenols, flavonoids, terpenoids, alkaloids, and proteins, play crucial roles in reducing metal ions and stabilizing nanoparticles, thereby eliminating the need for harsh chemical catalysts [12–14]. This technique is widely regarded as a clean technology as it significantly reduces the generation of toxic waste, occupational risks, and environmental impacts [13–15]. Furthermore, green-synthesized nanoparticles demonstrate enhanced biocompatibility, improved bioavailability, and reduced cytotoxicity, which broadens their applicability in fields such as dental biomaterials [16–17]. Owing to their high adaptability to various metals, including silver, zinc, iron, and platinum, their operational simplicity, and the ability to control nanoparticle size and morphology by selecting plant extracts and reaction conditions, green synthesis is gaining increasing prominence in health-related nanotechnology [8,10]. It is considered a promising approach that integrates technological innovation, biological safety, and environmental responsibility [8,10,16].

An interesting example are silver nanoparticles, particularly those synthesized via green methods, which have recently become the focus of significant attention [18–19]. The eco-friendly applications of AgNPs in the biomedical, pharmaceutical, cosmetic, sanitation, and electronic sectors have driven extensive research into their biosynthesis [20–22]. Silver nanoparticles exhibit unique physical and chemical properties that enhance their versatility across multiple applications [23–25]. Biosynthesized AgNPs have been assessed regarding their antimicrobial, antioxidant, and anticancer effects, as well as for their therapeutic potential in treating dermatitis and other conditions [26]. Studies have demonstrated that these nanoparticles exhibit low toxicity, effectiveness against antibiotic-resistant microorganisms, and strong colloidal stability, ensuring long-term dispersion [26–27]. Additionally, they exhibit antioxidant activity and selective cytotoxic effects on tumor cells, including oral cancer cell lines [24,28]. In dentistry, green-synthesized nanoparticles have been explored in various clinical applications. They are effective in preventing biofilm formation and growth of key oral pathogens, such as *Streptococcus mutans* and *Candida albicans*, particularly when incorporated into dental materials or applied to prosthetic and restorative surfaces [8,25–26].

Despite the potential for application in dentistry, there is a lack of reviews integrating recent advances in the green synthesis of metallic nanoparticles in this field. Therefore, this scoping review employed the “population, concept, context” (PCC) strategy to explore how these metallic nanoparticles (population) obtained through green synthesis (concept) are being applied in dentistry (context).

## Review

**Figure 1:**
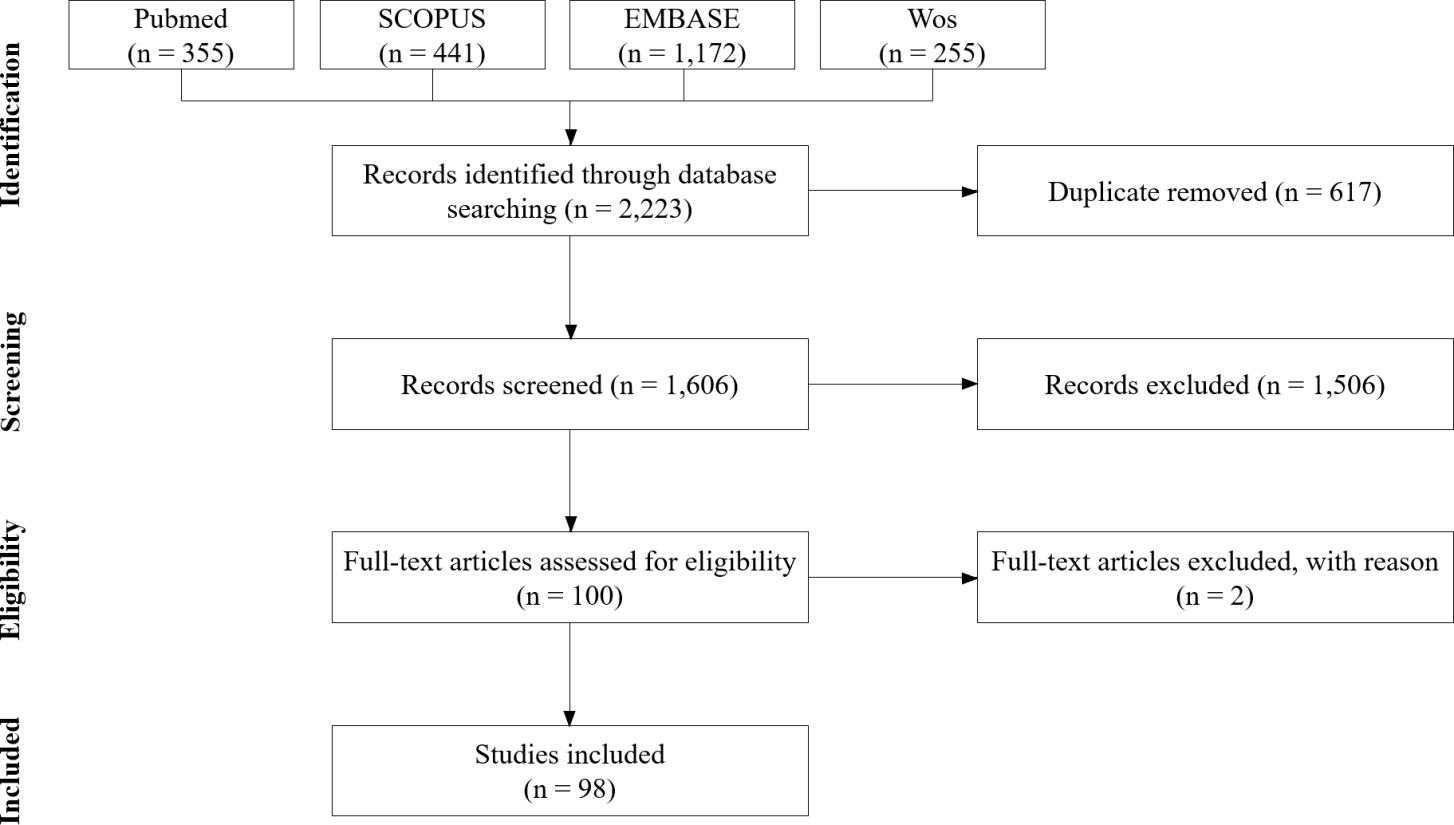
Flowchart summarizing the data management process to obtain the study articles.

We identified 1606 non-duplicate articles, from which 1,506 articles were excluded for not meeting the eligibility criteria. The full texts of 100 articles were assessed, with two further exclusions due to insufficient methodological data. The final sample consisted of 98 articles included for qualitative synthesis (Figure 1).

### Where and when are articles from?

**Figure 2:**
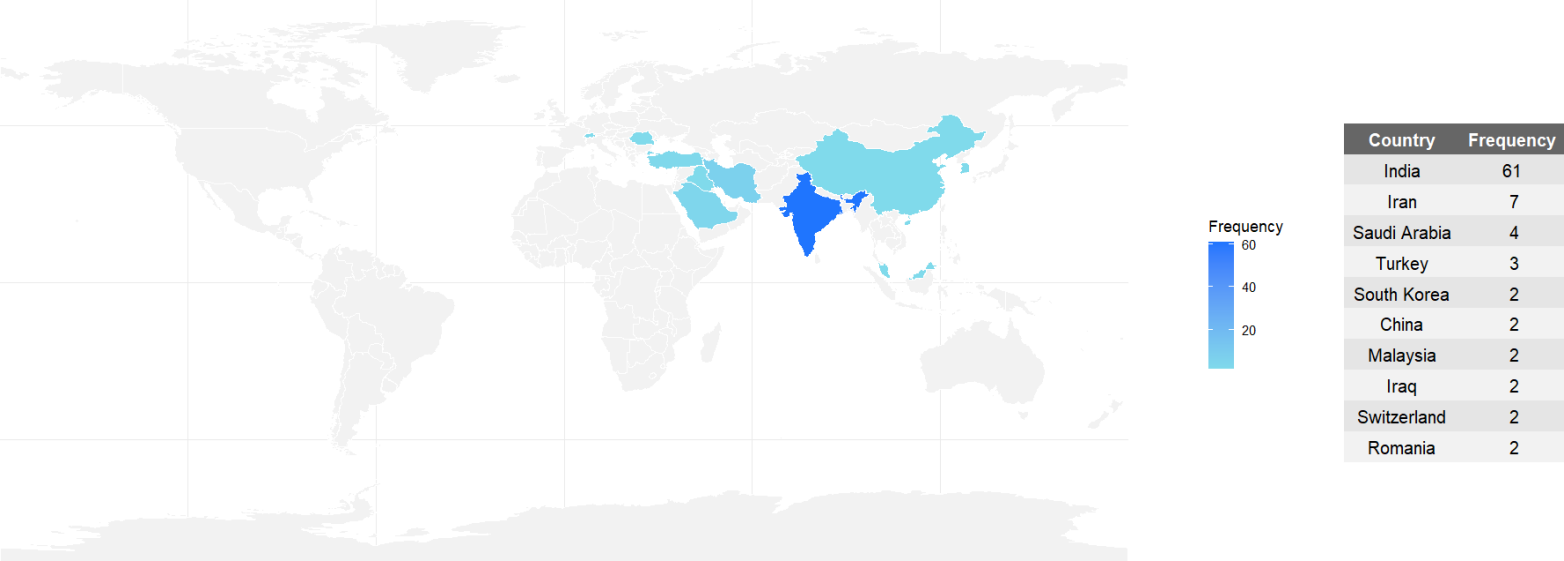
Number of publications about green-synthesized metallic nanoparticles by country.

Of the 98 articles selected for this review, 77 (78.6%) originated from India (Figure 2). It is important to highlight that the combined number of publications from all other countries does not surpass India’s contribution to this topic.

The predominance of India in the number of published articles related to the green synthesis of metallic nanoparticles in dentistry can be attributed to a combination of scientific, cultural, and socioeconomic factors. First, India possesses a rich biodiversity, with a variety of endemic medicinal plants traditionally used in Ayurvedic and Unani medicine [10]. The country’s long-standing cultural familiarity with plant-based therapeutics provides a robust foundation for research into botanical extracts as reducing and stabilizing agents in nanoparticle synthesis [29].

Additionally, India has significantly invested in scientific research over the past decades, particularly in nanotechnology, biotechnology, and pharmaceutical sciences. The country is home to a large number of public and private universities, research institutes, and government-funded agencies (e.g., “Council of Scientific & Industrial Research” (CSIR), “Indian Council of Medical Research” (ICMR), and “Department of Biotechnology” (DBT)), which actively promote low-cost, eco-friendly innovation, particularly in healthcare [30–31]. The economic feasibility and simplicity of green synthesis methods also align well with the resource-limited infrastructure often encountered in academic laboratories across developing nations.

India’s high burden of oral and systemic infectious diseases, particularly in underserved populations, has also contributed to increased research efforts seeking affordable and sustainable alternatives to conventional antimicrobial therapies. In this context, the green synthesis of metal nanoparticles becomes a strategic research focus that addresses local public health needs while offering potential for low-cost translational applications [32]. In summary, India’s leadership in this domain is the result of an interplay between cultural heritage, biodiversity, public health priorities, institutional infrastructure, and scientific dissemination practices, which together create fertile ground for prolific research on green-synthesized nanoparticles in dentistry.

**Figure 3:**
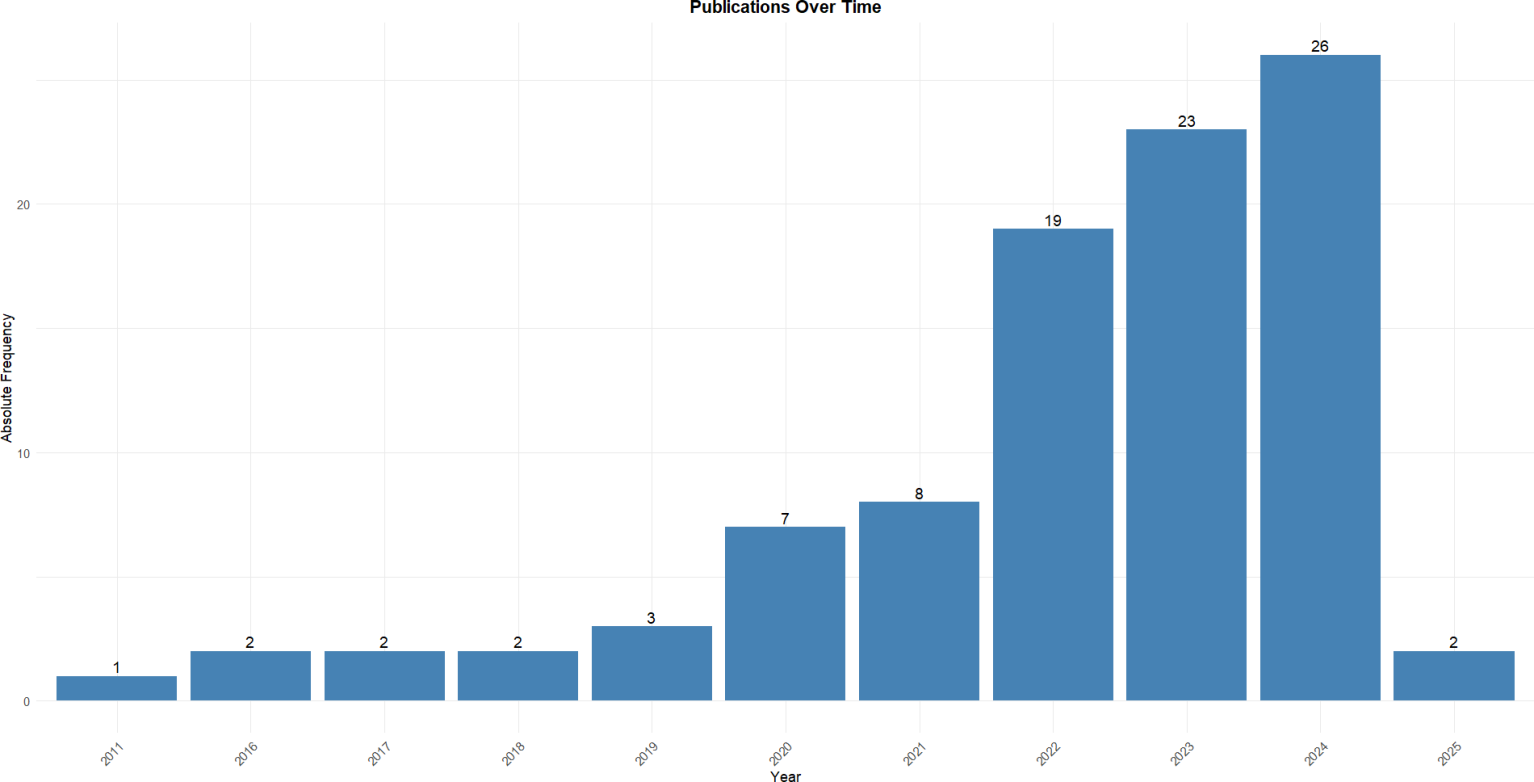
Publications in green-synthesized metallic nanoparticles used in the dental field over the years (2011–2025).

It is also important to highlight that, throughout the 2020s, studies involving metallic nanoparticles obtained via green synthesis in dentistry have shown a consistent year-by-year increase (Figure 3). This increase may be primarily related to the recent pursuit of sustainable and biocompatible methods.

### Plants, metals, and methods used in green synthesis

**Figure 4:**
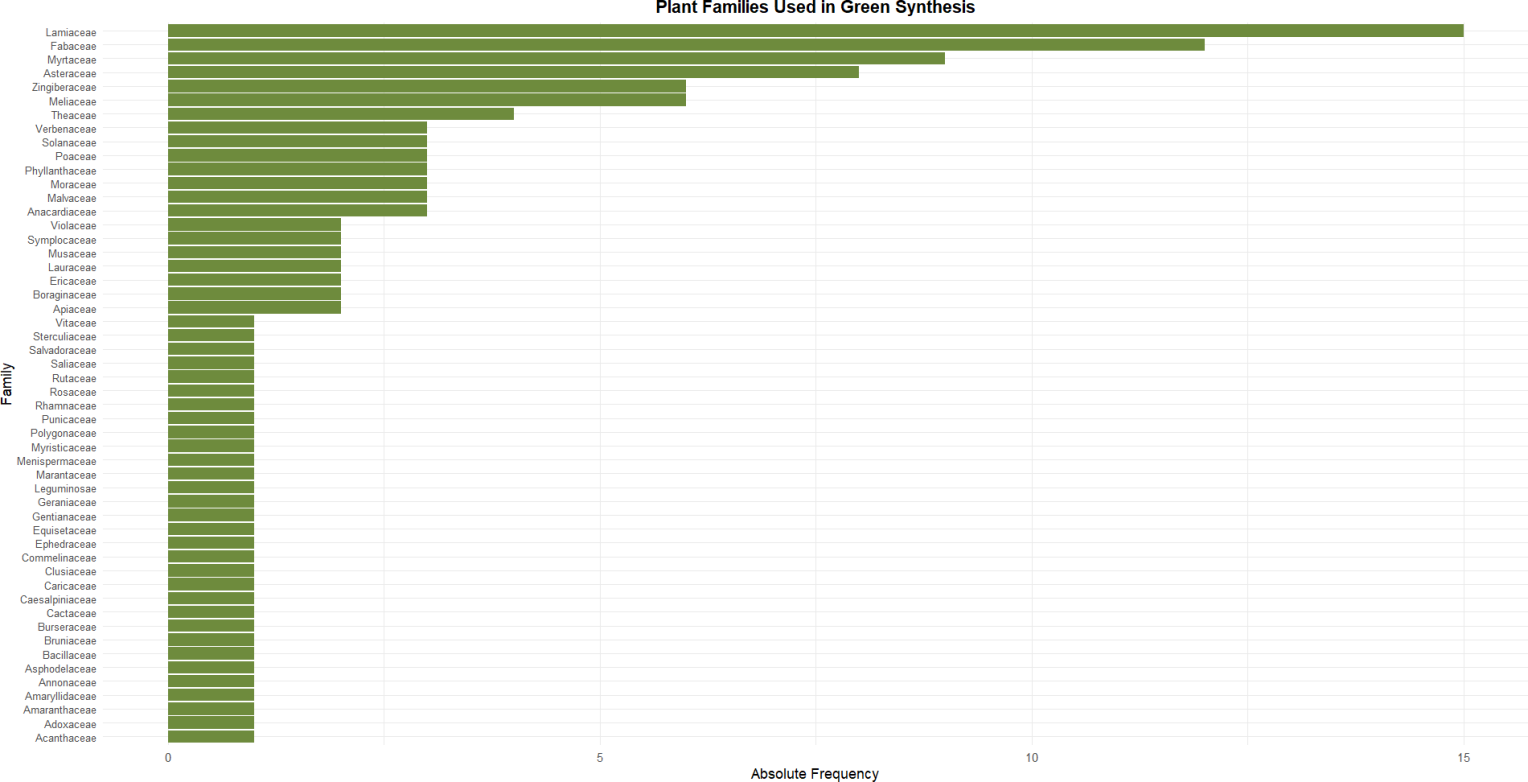
Botanical families used in green-synthesized metallic nanoparticles used in dentistry.

The predominance of plant families such as Lamiaceae (*n* = 15), Fabaceae (*n* = 12), Myrtaceae (*n* = 8), Asteraceae (*n* = 7), and Zingiberaceae (*n* = 6) among the species used in green synthesis (Figure 4) reflects their rich phytochemical profiles, particularly their high content of phenolic compounds [33]. These secondary metabolites, including flavonoids, tannins, phenolic acids, and terpenoids, act as both reducing and stabilizing agents during the biosynthesis of metallic nanoparticles [34–35].

For instance, plants from the Lamiaceae family (e.g., *Ocimum* spp., *Rosmarinus officinalis,* and *Salvia rosmarinus*) are extensively documented for their abundant polyphenols such as rosmarinic acid and caffeic acid, which facilitate the reduction of metal ions and promote the nucleation and capping of nanoparticles [36–37]. Similarly, species within the Fabaceae family (e.g., *Glycyrrhiza glabra*, *Clitoria ternatea*, and *Cassia fistula*) produce significant quantities of isoflavones and tannins that contribute to controlled nanoparticle morphology and size, which are crucial for optimizing biological activity [8,38]. The Myrtaceae family, particularly *Syzygium aromaticum*, is a well-established source of eugenol, a potent phenolic compound with known antimicrobial and antioxidant properties, enhancing the bioactivity of synthesized nanoparticles [39–40]. The Asteraceae family includes species rich in flavonoids like quercetin and luteolin, and sesquiterpene lactones, which have been associated with both nanoparticle synthesis and broad-spectrum biological effects, such as antibiofilm and anticancer activities [36,41]. Plants from the Zingiberaceae family, especially *Zingiber officinale*, provide gingerols and shogaols, phenolic compounds known not only for their anti-inflammatory and antioxidant effects, but also for their ability to support nanoparticle synthesis under mild conditions [42].

This review identified that two botanical families, Fabaceae and Lamiaceae, stood out as recurrent sources of phytochemicals used in the green synthesis of metallic nanoparticles [9,43]. Nanoparticles derived from these families have been incorporated into dental applications such as restorative materials and endodontic medicaments. In cariology, plant-mediated AgNPs and ZnO-NPs demonstrated significant antibacterial activity against *Streptococcus mutans* biofilms [8–9,43], whereas in endodontics, their integration into irrigants and sealers improved disinfection efficiency against *Enterococcus faecalis* [44]. These findings underscore the translational potential of plant-based nanotechnology in core areas of dentistry.

The high frequency of these families in the reviewed articles indicates a strategic phytochemical selection that maximizes both synthetic feasibility and therapeutic potential. This correlation highlights the critical role of phenolic-rich botanical species in enabling the eco-friendly, scalable production of biofunctional nanoparticles suitable for applications in oral health, including antimicrobial, antibiofilm, and tissue-regenerative uses.

A consistent observation across the reviewed studies was the superior biological behavior of nanoparticles synthesized through green chemistry compared to those obtained via conventional methods. This enhanced activity is frequently attributed to the presence of plant-derived bioactive compounds, such as flavonoids, polyphenols, and terpenoids, that act as both reducing and capping agents [13]. These phytochemicals not only facilitate the formation of stable colloidal nanostructures but also contribute synergistically to their bioactivity, enhancing their interaction with microbial membranes and disrupting biofilm formation [10,24].

**Figure 5:**
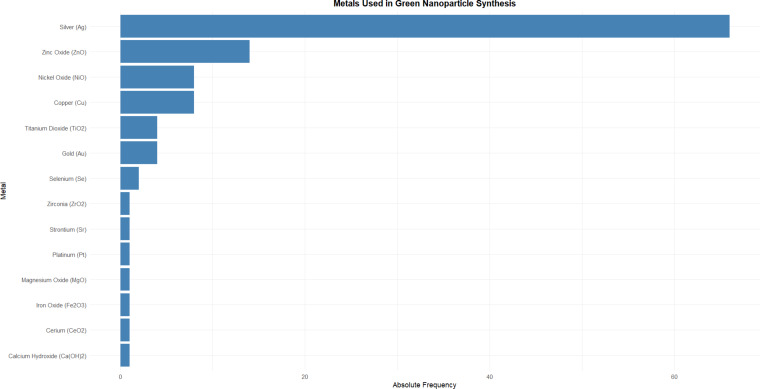
Metals used in nanoparticles green synthesis in the dental field.

In this study, silver (66%) was the main metal used in nanoparticle synthesis with an eco-friendly approach (Figure 5). The wide predominance of AgNPs can be attributed to their well-documented antimicrobial, antifungal, and anti-inflammatory properties, making them especially attractive for applications in dental materials aimed at infection prevention and biofilm control [45–46].

Besides silver, zinc, copper, and nickel, other metals have been used to a lesser extent. The significant representation of ZnO-NPs, known for their biocompatibility, UV-blocking capacity, and antibacterial efficacy, aligns with their growing use in restorative and preventive dentistry, including cements, sealants, and mouthwashes [8,45]. CuNPs and nickel oxide nanoparticles, though less frequent, are also gaining attention for their antimicrobial activity, despite some concerns regarding cytotoxicity [46–47]. The presence AuNPs and titanium dioxide nanoparticles highlights an interest in optical and photocatalytic applications, particularly in regenerative procedures and dental coatings [48–49]. The sparse use of other metals such as magnesium, iron, selenium, and cerium oxides suggests ongoing exploration of less conventional materials with niche properties, possibly linked to targeted therapeutic functions or synergistic effects when combined with more established agents [50–51]. Overall, the choice of metal reflects a balance between desired biological activity, safety profile, and the feasibility of green synthesis, all critical for clinical translation in dental practice.

We highlighted that 68.80% (*n* = 51) of the studies describing nanoparticle synthesis methods employed stirring. Other reported methods included heating (27.30%; *n* = 21) and cooling (3.90%; *n* = 3). Stirring is a widely used method in the green synthesis of metallic nanoparticles because it plays a crucial role in ensuring efficient nucleation, growth, and stabilization of nanoparticles. Once plant extracts are commonly used as reducing and capping agents, stirring promotes the homogeneous mixing of the metal ions and the bioactive compounds in the extract. This enhances the frequency of collisions between them and results in more uniform nucleation and better control over particle size and morphology. Moreover, continuous stirring prevents aggregation of the nanoparticles, contributing to their colloidal stability. Studies have shown that variations in stirring speed can significantly impact the yield and characteristics of the resulting nanoparticles, making it an essential parameter for optimizing reproducibility and ensuring eco-friendly synthesis processes [52–53].

### Metallic nanoparticles characterization

**Figure 6:**
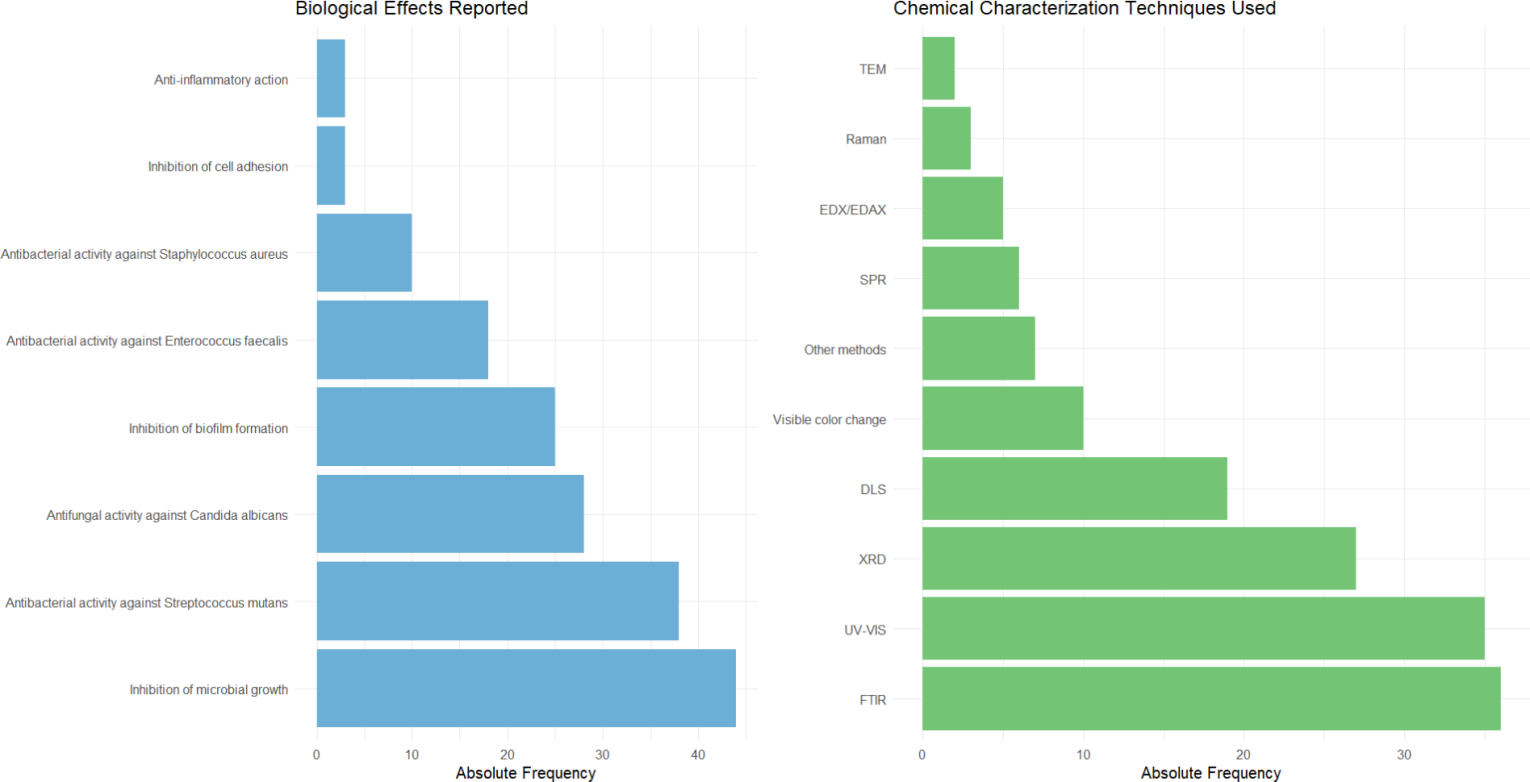
Biological and chemical characterization of nanoparticles used in the selected studies.

The studies characterize the nanoparticles obtained through green synthesis in terms of both physicochemical properties and biological effects (Figure 6). The most frequently reported biological activity among the selected studies was the inhibition of microbial growth (80.0%; *n* = 44), highlighting its prominence as a primary target in antimicrobial research. The studies primarily evaluated antimicrobial effects against *Streptococcus mutans* (69.10%; *n* = 38), *Candida albicans* (50.90%; *n* = 28), and *Enterococcus faecalis* (32.70%; *n* = 18), demonstrating the broad versatility of green-synthesized metallic nanoparticles in targeting various biofilm-associated oral diseases [54–55]. The investigation of antimicrobial activity against these microorganisms indicates the potential dental applications of green-synthesized metallic nanoparticles.

To characterize the green-synthesized metallic nanoparticles, the selected studies used mainly Fourier-transform infrared spectroscopy (FTIR, 36.73%; *n* = 36), ultraviolet–visible spectroscopy (UV–vis, 34.69%; *n* = 35), and X-ray diffraction (XRD, 25.48%; *n* = 27). They are among the most commonly used techniques for the characterization of metallic nanoparticles synthesized via green routes due to their complementary abilities to elucidate key structural, optical, and chemical properties. FTIR enables the identification of functional groups involved in the reduction and stabilization of nanoparticles, typically derived from phytochemicals in plant extracts used as reducing agents [52,56]. UV–vis spectroscopy is widely employed to monitor nanoparticle formation in real time by detecting surface plasmon resonance bands, which provide insight into particle size and distribution [57]. XRD offers detailed information on the crystalline structure and phase composition of the nanoparticles, confirming successful synthesis and purity [53]. Together, these techniques form a robust analytical toolkit that supports the eco-friendly and scalable production of metal nanoparticles in green nanotechnology.

### Limitations concerning selected studies

The analysis of study limitations revealed that the vast majority (68.7%; *n* = 67) of the reviewed articles conducted exclusively in vitro experiments, with no progression to in vivo experimentation in 53.7% (*n* = 53) of cases. Moreover, 47.8% (*n* = 45) lacked cytotoxicity assessments or evaluations in human cells. This gap raises important concerns regarding the long-term biocompatibility, biodistribution, and potential cytotoxicity of these nanoparticles within the complex and dynamic oral environment [15,20]. A significant number of selected studies (*n* = 23; 24.1%) also failed to perform advanced physicochemical characterizations, such as FTIR, SEM, or XRD, which are essential for confirming nanoparticle properties.

Only a minority of studies investigated mechanisms of action (18.9%; *n* = 16) or addressed the scalability and reproducibility of the synthesis process (17.6%; *n* = 15), which are crucial for industrial and clinical translation. Comparisons with conventional treatments or commercial products were absent in 16.2% (*n* = 14) of the works, while 9.5% (*n* = 8) did not replicate their experiments. Limitations also included restricted animal modeling (8.1%; *n* = 7) and absence of long-term effect assessments (8.1%; *n* = 7).

Altogether, these findings indicate that while green-synthesized nanoparticles hold great promise for dental applications, there is still a substantial need for methodological thoroughness, translational progression, and standardized protocols to advance their clinical implementation and proper systematic efficacy comparisons. These limitations are herein represented in the variability of plant sources, extraction methods, synthesis parameters, and nanoparticle characterization techniques.

### Applications in the dental science field

Preventive and restorative dentistry represents the field that most extensively utilizes green synthesis of nanoparticles (Table 1), particularly in the development of antimicrobial compounds such as oral antiseptics and toothpastes with prolonged antimicrobial action, restorative materials (including composite resins and cements) with antibacterial properties, nanoparticle-reinforced sealants and fluoride varnishes for caries prevention, and antibiofilm coatings for dental and orthodontic surfaces.

**Table 1:** Summary of green-synthesized nanoparticle applications in dentistry.

Dentistry area	Applications with green synthesis of nanoparticles

preventive and restorative dentistry	mouthwashes [4,12–13,15–16,20,41,44,60–61], toothpastes [15,20,44,58–59], composites [61]
endodontics	irrigants [15,61–63], filling [62], intracanal medicament [4], nonspecific [64–65]
periodontics	gels [66], systems with controlled release of nanoparticles [67], dental floss [68], nonspecific [69–71]
implantology	coating of implants with nanoparticles [72], nonspecific [59]
orthodontics	nonspecific [15,59,72], orthodontic wire [73], brackets with antimicrobial coatings [19]

Beyond the initial findings, nanoparticles have also been incorporated into a wide range of preventive and restorative dental materials. Composite resins and glass ionomer cements reinforced with silver or zinc oxide nanoparticles exhibit enhanced antimicrobial activity, reducing bacterial colonization at restoration margins and thereby minimizing secondary caries risk [9]. Additionally, fluoride varnishes and dental sealants containing biogenic nanoparticles demonstrate antibiofilm effects and prolonged ion release, making them promising adjuncts in caries prevention strategies, especially in high-risk populations [8].

Within the dental sciences, green-synthesized nanoparticles have demonstrated considerable potential across multiple domains. In cariology and periodontology, these nanoparticles have been effectively incorporated into toothpastes, mouthwashes, and composite resins to inhibit or reduce microbial colonization by key oral pathogens such as *Streptococcus mutans*, *Lactobacillus* spp., and *Candida albicans* [27–28]. Their antimicrobial efficacy contributes to disrupting biofilm formation and controlling infection processes fundamental to dental caries and periodontal disease progression.

In periodontal therapy, green-synthesized nanoparticles have been formulated into bioadhesive gels, local delivery systems, and coated dental flosses to sustain antimicrobial release in periodontal pockets [18]. Such approaches improve the control of periodontopathogens such as *Porphyromonas gingivalis* and *Aggregatibacter actinomycetemcomitans*, reducing inflammation and supporting adjunctive treatment to scaling and root planing. Regenerative strategies are also being developed, in which nanoparticle-functionalized membranes have shown potential to modulate host immune response and stimulate periodontal tissue repair [72].

In the field of endodontics, biosynthesized AgNPs and ZnO-NPs have been employed as adjuvants to conventional irrigants and intracanal dressings, thereby enhancing the disinfection efficacy of root canal systems [21]. The nanoscale properties of these particles facilitate deeper penetration into complex canal anatomies and improve antimicrobial action against resistant endodontic pathogens, which is critical for preventing reinfection and ensuring treatment success.

The incorporation of biogenic nanoparticles into irrigating solutions, intracanal medicaments, and filling materials enhances their penetration and antibacterial action against resistant microorganisms, including *Enterococcus faecalis* [8,43–44]. Green-synthesized AgNPs and ZnO-NPs are particularly relevant due to their ability to disrupt biofilm architecture within dentinal tubules [8,43]. Moreover, their use in nanoparticle-coated gutta-percha cones has been proposed to improve long-term disinfection and reduce reinfection risks [44].

Furthermore, within dental tissue engineering, recent studies have reported promising outcomes using biogenic nanoparticles incorporated into scaffolds and regenerative membranes designed to stimulate osteogenesis and promote periodontal tissue regeneration [16–17]. These nanoparticles not only provide antimicrobial protection but also actively modulate cellular behavior, such as proliferation and differentiation, thereby enhancing the regenerative potential of biomaterials applied in clinical settings.

Implantology is another emerging area of application, where nanoparticle-based coatings are used to reduce peri-implant biofilm formation and enhance osseointegration [9,19]. Titanium implants functionalized with green-synthesized AgNPs or iron oxide nanoparticles doped with silver (Ag-Fe_2_O_3_) exhibited dual properties, that is, antimicrobial protection and stimulation of osteoblastic activity. In prosthodontics, biogenic nanoparticles have been incorporated into denture base resins and soft liners, demonstrating significant antifungal activity against *Candida albicans*, which is crucial for denture stomatitis management [18,59].

In orthodontics, nanoparticles have been applied as antimicrobial coatings on brackets and archwires to mitigate plaque accumulation and white spot lesions during treatment. Additionally, mouth rinses and gels containing biogenic AgNPs are being investigated as adjunctive therapies to improve oral hygiene in patients with fixed orthodontic appliances [8,37,44]. These strategies aim to balance microbial control while preserving the mechanical performance of orthodontic devices.

Green-synthesized nanoparticles also hold promise in regenerative dentistry. Incorporated into scaffolds, membranes, and hydrogels, they have demonstrated the ability to enhance osteogenesis and angiogenesis while simultaneously reducing microbial contamination [19]. These multifunctional biomaterials not only support bone and periodontal regeneration but also represent sustainable alternatives for next-generation regenerative therapies [8].

## Conclusion

In conclusion, this review emphasizes the growing significance of green nanotechnology in dentistry while underscoring the imperative for interdisciplinary collaboration and stringent regulatory oversight. The prominent use of silver and zinc oxide nanoparticles, produced through environmentally sustainable phytochemical-mediated synthesis, underscores a convergence of advanced antimicrobial functionality, enhanced biocompatibility, and alignment with global sustainability goals. These findings affirm that green nanotechnology represents a paradigm shift in dental material innovation, offering promising avenues to enhance oral health outcomes while integrating ecological responsibility. Future research must prioritize translational approaches, including comprehensive clinical trials and toxicological assessments, to validate the safety and efficacy of these innovative materials and facilitate their responsible integration into routine dental practice, as green-synthesized nanoparticles are pioneering agents on the verge of redefining the future of dental therapeutics and biomaterials.

## Review Guidelines

### Review protocol and objective

This scoping review followed the PRISMA guidelines for systematic reviews and was registered in “Open Science Framework” (https://osf.io/n3htg/). The main objective was to identify and analyze studies on the application of metallic nanoparticles synthesized via green chemistry in dentistry.

### Search strategy and information sources

This review was guided by the PCC framework, that is, population (P): metallic nanoparticles (e.g., silver, gold, copper, zinc oxide); concept (C): green synthesis using plant-based extracts or eco-friendly biosynthetic routes; context (C): dental applications (e.g., oral microbiology, materials science, cariology, periodontology, endodontics). Thus, we used a combination of Boolean expressions that correspond to the PCC (Table 2).

**Table 2:** Boolean expression used in this scoping review.

Category	Keywords / Terms

population	metal OR metallic OR ZnO OR Gold OR Silver OR Copper OR Cobalt OR Niquel AND nanoparticle
concept	"biosynthesis" OR "green synthesis" OR ecofriendly OR "eco-friendly" OR "plant extracts" OR "herbal extracts" OR "phytochemicals"
context	dentistry OR mouth OR oral OR teeth OR tooth OR tongue OR odontology OR 'oral science'

### Eligibility criteria

Inclusion criteria were original research articles evaluating the use of green-synthesized metallic nanoparticles in dental contexts, articles published in English, studies with experimental or in vitro/in vivo design, full publication until May 2025, and peer-reviewed publications. Exclusion criteria included reviews, editorials, letters to the editor, conference abstracts, and studies not focused on dentistry.

### Study selection and data management

A total of 2,223 articles were retrieved (PubMed = 355; Scopus = 441; Embase = 1,172; Web of Science = 255). After removal of 617 duplicates using Rayyan QCRI (RRID:SCR_017584), 1,606 studies were screened based on titles and abstracts by two independent reviewers.

### Data extraction

Data were extracted regarding authorship, publication year, country, type of metal used, part of the plant, synthesis method, nanoparticle characterization techniques, biological evaluations, and application within dentistry.

## Data Availability

Data sharing is not applicable as no new data was generated or analyzed in this study.
